# Strike's Effect on Health

**Published:** 1920-10-23

**Authors:** 


THE PUBLIC WELL-BEING—(*continued*).

### Strike's Effect on Health.

The coal strike is a very serious fact in every respect, and one of the most serious consequences will be its influence upon the nation's health. The effects will be direct as well as indirect. It will be direct, first of all, upon the many thousands who will be without employment and without sufficient means to buy what is necessary to maintain fitness, added to which will be the mental worry which has so definite a bearing upon physical well-being. Upon the general mass of the community, and more especially upon those less well-to-do, the strike will fall heavily. It comes at the worst time of the year, when the warm weather has gone. Restriction in food and the purchase in thousands of cases of the cheapest and nastiest varieties are other debilitating factors, and the whole effect of the strike is to lower the general physical tone of great numbers of people. The old, the young, and the sick will be the first to suffer. Every mother will be anxious about the supplies of milk for her children. Every hospital, every nursing home, every institution which tends the sick and infirm will feel the pinch now and for a long time afterwards. The ground will be carefully prepared for the havoc of epidemics, such as those of influenza, because the power of physical resistance to disease will be widely lowered. It is obvious that if at the end of the struggle the miners get their two shillings they will have lost much more than the two shillings, and everybody else will have lost with them. It is also obvious in the more than probable event of the miners failing to achieve their ends, that everybody will again be the loser. Everybody stands to lose in either event, and for this the country is to go through a period of loss of material prosperity, of lowering of resistance to disease, of suffering, and anxiety. Napoleon flattered us when—and if—he called us a nation of shopkeepers, for a large section of us, at any rate, does not now appear to understand the simple elements of a profit and loss account. It is absurd to pretend to argue that a strike in such a fundamental industry as coal, and in such circumstances as the present strike, is not a harsh and cruel expedient. It is almost enough to dishearten every zealous worker for an improved national health record and better social conditions. It sets back seriously all sustained efforts towards the mitigation of disease, and it makes more difficult than before the uphill task of securing funds for all the humane agencies like the hospitals and other such institutions and organisations. The good things of life can never be won by destroying them. Let us hope wiser counsels will prevail before things become any worse than they now are.

*The Times* medical correspondent reports a meeting of the Federation of Medical and Allied Societies to appoint a sub-committee to take action on behalf of the sick and invalid during the strike. The Federation co-operated with the Royal Society of Medicine during the railway strike to secure transport in necessary cases. It represents all phases of medical activity, from doctors to nurses, and consequently feels that it can do much towards securing that whoever may suffer those in the hands of the medical profession by reason of their infirmities shall not.

